# A Child’s Teeth

**Published:** 1885-02

**Authors:** W. Geo. Beers

**Affiliations:** Montreal


### A CHILD'S TEETH.

BY W. GEO. BEERS, L. D. S., MONTREAL.

For many years I have been specially interested in the study of the predisposing causes of caries in children's teeth. It is a subject we can never investigate sufficiently, until we stop guessing and are able with certainty to tell why *decay during growth* should confine itself to the teeth as a rule, and until we know what mother and child are to do in self-defence. I might repeat a good deal I have said elsewhere, but among a large record of cases faithfully examined, and whose family history I have endeavored to trace, I have selected one of the very worst cases I ever met, that of a young girl aged twelve years.

The following description will hardly speak for itself, as it does not tell of the structural poverty, the thinness of enamel, the almost plaster of paris character of the very dentine.

In the superior teeth there are twenty-three fillings upon all the different surfaces of the teeth. Some of the cavities are of the most extensive character, involving nearly the whole of the crown. There is but one undecayed tooth, a cuspid, and this gives indications of approaching disease. In the inferior teeth there are fourteen fillings, with but four sound teeth, and these only comparatively so. Thus there are found in the twenty-eight teeth of this patient thirty-seven distinct cavities.

The mother of the child recently died, but from the father I obtained the following particulars : The mother did not suffer from any disease during pregnancy, except the ordinary attacks of nausea, so common at that time. These lasted almost six weeks, but did not cause as much suffering as during a former pregnancy. The mother's health was never very good. My little patient was quite free from the usual diseases of children until the winter of '78-'79, when she was seven and one-half years old. She then had scarlet fever. About three or four years ago she had measles. The parents have been very careful never to overtax her physical or mental strength, and everything that attention and care could accomplish for her was always done.

On her mother's side the family history of health is not good. The mother very nearly died of peritonitis when the first child was born. Seventeen months after, my patient was born, and the mother was able to nurse her, and was in the enjoyment of rather better health than she ever had before or since. The first child died in October (1871), after my patient's birth. Two other children died, each a few months old, one in 1873, the other in 1875. The mother died, after a prolonged illness, of asthma, last year. The grandmother on the mother's side was a confirmed invalid for a great many years. Her teeth were never good, owing, it was thought, to excessive doses of calomel, etc. The great-grandmother on the mother's side also suffered for many years from asthma, though she lived to old age. On the mother's father's side the antecedent's are good, and also on the side of the father.

Now, I think in tracing family history in this way we get to the only true—the embryonal—cause of the extraordinary condition of my little patient's teeth. Through the blood of the mother the teeth of the child are influenced. The more I study cause and effect in this connection, the more impressed I am that we want a new departure in physiological science; the birth of a new branch of medicine and dentistry almost exclusively devoted to embryology, that will educate the public to confide in it, or be advised by it. The mere filling of carious cavities in children's teeth, or even the administration of easily assimilated phosphates, or the direction of proper diet, are all on the late side. Family history must be traced before pregnancy if possible, and the embryologist must tell us what is to be done to divert hereditary tendencies, and to improve the prospects of the coming embryo.

